# Comments on ”Evidence of the hydrogen release mechanism in bulk MgH_2_”

**DOI:** 10.1038/srep44216

**Published:** 2017-04-07

**Authors:** Alexander Surrey, Kornelius Nielsch, Bernd Rellinghaus

**Affiliations:** 1 IFW Dresden, Institute for Metallic Materials, Dresden, D-01171, Germany; 2 Technische Universität Dresden, Institut für Festkörperphysik, Dresden, D-01062, Germany

## Abstract

The effect of an electron beam induced dehydrogenation of MgH_2_ in the transmission electron microscope (TEM) is largely underestimated by Nogita *et al*., and led the authors to a misinterpretation of their TEM observations. Firstly, the selected area diffraction (SAD) pattern is falsely interpreted. A re-evaluation of the SAD pattern reveals that no MgH_2_ is present in the sample, but that it rather consists of Mg and MgO only. Secondly, the transformation of the sample upon *in-situ* heating in the TEM cannot be ascribed to dehydrogenation, but is rather to be explained by the (nanoscale) Kirkendall effect, which leads to the formation of hollow MgO shells without any metallic Mg in their cores. Hence, the conclusions drawn from the TEM investigation are invalid, as the authors apparently have never studied MgH_2_.

The investigation of MgH_2_ by transmission electron microscopy (TEM) is challenging due to the high sensitivity of the material to high energy electrons. It is well known that MgH_2_ decomposes to Mg in the TEM within seconds due to radiolysis^1^,^2^,^3^,^4^,^5^,^6^,^7^. This effect is largely underestimated by Nogita *et al*.^8^ which led the authors to a misinterpretation of their experimental obervations. Electron-beam induced heating of the sample is indeed negligible as discussed by the authors. The dehydrogenation of MgH_2_, however, is caused by radiolysis rather than heating^1^.

## Re-evaluation of SAD pattern

**Figure 1:**
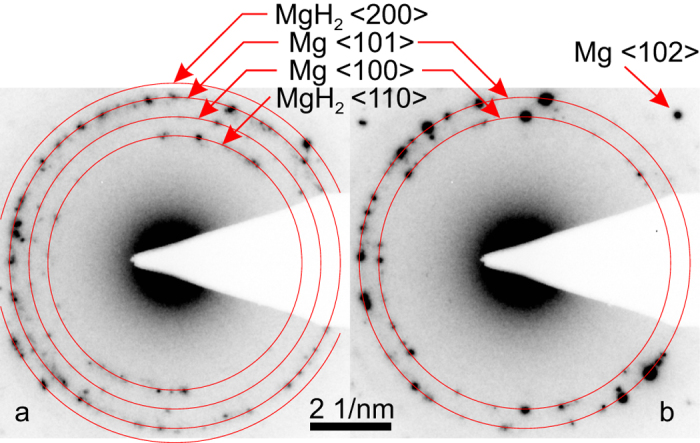
SAD patterns of a nanocrystalline MgH_2_ particle. The pattern in (**a**) was recorded under low dose conditions after irradiating the sample area for no longer but a few seconds. It shows both Mg and MgH_2_ diffraction spots. (**b**) After an exposure over 60 s, all MgH_2_ diffraction spots have vanished, and new Mg diffraction spots occur in the pattern.

In order to scrutinize the conclusions drawn in the paper, we have firstly re-evaluated the SAD pattern in Fig. 1 of the paper^8^. Since no scale bar is presented, we have used the Mg diffraction spots for calibration of the pattern. The resulting lattice plane spacings *d*_*m*_ are summarized in Table 1. The data clearly reveal that those diffraction spots which were identified by Nogita *et al*.^8^ as originating from MgH_2_ deviate by up to 16% from values *d*_*t*_ that can be found in the literature for *α*-MgH_2_ (rutile structure)^9^. In contrast to the conclusion drawn by Nogita *et al*.^8^, this suggests that there is *no* MgH_2_ present in the specimen. The diffraction spots rather are to be attributed to MgO, for which the measured values agree perfectly with the tabulated data (cf. Table 1). It thus is to be concluded that the authors have studied MgO rather than MgH_2_, while the latter is most likely decomposed under the influence of the electron beam already during the alignment of the sample prior to the measurement.

## Electron beam induced dehydrogenation of MgH_2_ monitored by electron diffraction

In order to verify the result of our re-evaluation, we have repeated the electron diffraction experiment on high-energy ball milled MgH_2_, which was prepared and handled under argon. The sample was exposed to air only for a few minutes during the transfer into the TEM. From X-ray diffraction it is known that the as prepared ball milled material is originally fully hydrogenated and does not contain any metallic Mg. The SAD patterns shown in Fig. 1 were acquired on a probe and image C_s_ corrected FEI Titan^3^ 80–300 operated at 300 kV. It was only possible to record diffraction spots that correspond to MgH_2_ by massively reducing the electron current density to about 2500 electrons/s/nm^2^ and by avoiding any illumination of the specimen area of interest prior to acquiring the SAD pattern from this area. With the SAD aperture an area with a diameter of roughly 200 nm of the central part of the MgH_2_ particle is selected. In the SAD pattern in Fig. 1a many individual diffraction spots are visible, which stems from the nanocrystalline morphology of the sample. Nogita *et al*. measured much less diffraction spots, which is typical for single crystalline areas of the specimen. We, therefore, assume that the average grain size in their sample is generally larger than in the ball-milled MgH_2_ studied in the present work. The SAD pattern in Fig. 1a reveals both Mg and MgH_2_ diffraction spots. This shows that already the short and low dose exposure to the electron beam during adjusting the specimen for about 3 s caused a partial dehydrogenation. The intensity of the MgH_2_ diffraction spots decreases rapidly within seconds. At latest, after electron irradiation for 1 min, which corresponds to a maximum electron dose of 150 · 10^3^ electrons/nm^2^, the specimen is fully dehydrogenated, and no traces of MgH_2_ are present in the SAD pattern anymore (cf. Fig. 1b). Obviously, the preparation and thereby the morphology of the ball-milled MgH_2_ differs from that of the Mg-Ni-based hydrogen storage alloy, which is investigated by Nogita *et al*. However, the here discussed susceptibility to the electron beam can be considered to be comparable for both materials.

## *In-situ* TEM heating

**Figure 2:**
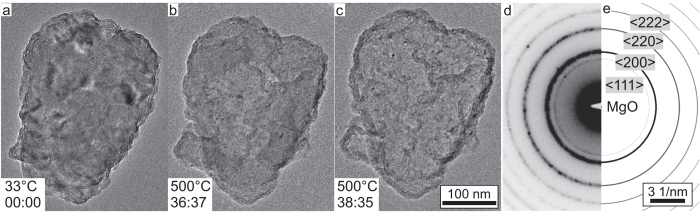
(**a–c**) Still frame TEM images. These images are selected from the *in-situ* heating video S1 (see Supplementary information) of the Mg particle that was obtained through electron beam induced dehydrogenation during the former acquisition of SAD patterns (cf. Fig. 1). Temperature and time stamp (format mm:ss) are indicated in the images, respectively. (**d**) SAD pattern of the sample at the end of the heating process at *T* = 500 °C. (**e**) Simulated electron diffraction ring pattern of MgO.

We have also conducted an *in-situ* TEM heating experiment comparable to the one reported by Nogita *et al*.^8^. For this, we have studied exactly the same meanwhile fully dehydrogenated MgH_2_ particle that was priorily studied by electron diffraction (cf. Fig. 1). For this experiment we have used the Wildfire S3 heating holder from DENS solutions. The constant heating rate of 13 K/min is the same as the one applied by Nogita *et al*.^8^. Figure 2 shows three still frame TEM images from the *in-situ* heating video S1 that can be found in the Supplementary information. The images show a relative high noise level because of the reduced electron current density that was used in order to again minimize any further impact of the electron beam. Figure 2a shows a TEM image of the particle at *T* = 33 °C. The relatively strong contrast variations within the particle indicate a high density of defects in the nanocrystalline Mg particle. Upon annealing the sample to *T* = 480 °C, the contrast variations are reduced due to partial recrystallization. This becomes also obvious from video S1. At *T* = 486 °C, a region of bright contrast begins to form and discontinuously grows in the centre of the particle. The temperature was then kept constant at *T* = 500 °C, thereby allowing this reaction to complete, which took about 2 min. Figure 2c shows the fully transformed particle, which is characterized by a much brighter contrast in the core and a shell with a dark contrast. This observation is identical to the results presented by Nogita *et al*.^8^, however, with the exception that in the present case, the transformation occurs at temperatures which are roughly 50–60 K higher than in the report of Nogita *et al*.^8^. This discrepancy can either be ascribed to the uncertainty with which temperatures can be measured in a TEM heating holder. It can, however, also be due to the impact of the electron beam (the dose rate of which must be clearly higher during the experiments of Nogita *et al*.^8^), which is known to promote solid state diffusion processes^10^.

We have finally acquired another SAD pattern of the fully transformed particle at *T* = 500 °C, which is shown in Fig. 2d. It perfectly agrees with the simulated MgO ring pattern displayed in Fig. 2e, which is calculated with the MacTempas software package^11^ using electron scattering factors. A comparison with commercial MgO nanopowder is given in Figure S2 in the Supplementary information, which again confirms that the SAD pattern shown in Fig. 2d can be undoubtedly attributed to MgO. The continuous and diffuse diffraction rings indicate a nanocrystalline microstructure. No other diffraction spots or rings are observed. From the particle morphology in the TEM images and the lattice spacings measured from the diffraction pattern, it can only be concluded that the particle is hollow and consists merely of a thin (10–20 nm) MgO shell.

It is well known that MgH_2_ is usually passivated by a thin surface oxide. This surface layer prevents the sample from further oxidation also for a short exposure to air of less than 5 min. Friedrichs *et al*. have investigated the oxidation behavior of nanocrystalline Mg and MgH_2_ prepared by ball milling and gas phase condensation using *in-situ* X-ray photoelectron spectroscopy and high-resolution TEM^3^. Both techniques are very surface sensitive and have revealed that all samples under investigation in this study show nanocrystalline oxide and amorhpous hydroxide layers with thicknesses of less than 4 nm even if the samples are handled under argon or high vacuum. Due to the low thickness and the low degree of crystallinity, this passivation layer is difficult to detect by X-ray or electron diffraction^3^. As a result, the surface oxidation of MgH_2_ is scarcely reported in the literature, especially for bulk samples, where a few nm thin oxide layer is not of great importance. This also explains why no diffraction spots or rings of MgO are observed in the SAD patterns in Fig. 1 prior to *in-situ* heating for the here studied MgH_2_. Additionally, the SAD patterns are acquired with a very low electron dose rate, which is why possible weak MgO reflections cannot be measured due to a too low signal-to-noise-ratio.

The Mg particle, which was formed through electron beam induced dehydrogenation on the TEM and which is more sensitive to oxidation than MgH_2_^3^, is then further oxidized at higher temperatures due to the residual oxygen and water in the high vacuum column of the TEM. As a result, the diffraction rings of the meanwhile somewhat thicker MgO surface layer is now clearly observable in the SAD pattern in Fig. 2d. The loss of the Mg core is attributed to the well known (nanoscale) Kirkendall effect, which causes an outward diffusion of Mg atoms, followed by their evaporation. This effect is frequently used to synthesize hollow metal-oxide nanoparticles by oxidation in air at elevated temperatures (see e.g. refs 12, 13, 14, 15, 16, 17). Krishnan *et al*. have also observed such an outward diffusion of Mg in nanoparticles, which was reported to result in hollow MgO nanoparticles^18^.

## Summary

In summary, there is only one possible conclusion that can be drawn: The sample investigated by Nogita *et al*.^8^ was *not* MgH_2_ at any time. Hence, the conclusions drawn from their TEM observations can only be invalid. As a consequence, any models based on them are to be considered speculative.

## Additional Information

**How to cite this article:** Surrey, A. *et al*. Comments on “Evidence of the hydrogen release mechanism in bulk MgH_2_”. *Sci. Rep.* **7**, 44216; doi: 10.1038/srep44216 (2017).

**Publisher's note:** Springer Nature remains neutral with regard to jurisdictional claims in published maps and institutional affiliations.

## Supplementary Material

Supplementary Video S1

Supplementary Information

## Figures and Tables

**Table 1:** Re-evaluation of the SAD pattern shown in Fig. 1 in ref. 8.

Structure	<hkl>	Re-measured *d_m_* (Å)	Tabulated *d*_*t*_ (Å)	Deviation^a^ (%)
Mg	<100>	—	2.78^b^	—
Mg	<2–10>	—	1.61^b^	—
*α*-MgH_2_	<10–1>	2.10	2.51	16.4
*α*-MgH_2_	<020>	2.10	2.26	7.1
*α*-MgH_2_	<12–1>	1.48	1.68	12.0
MgO	<200>	2.10	2.10	0.1
MgO	<220>	1.48	1.49	0.5
MgO	<212>	1.39	1.40	1.1

The Mg diffraction spots are used for calibration. All other diffraction spots can clearly be better attributed to MgO rather than to MgH_2_.

^a^The relative deviation is calculated from |*d*_*m*_ − *d*_*t*_|/*d*_*t*_.

^b^These reflections were used to calibrate the SAD pattern.
