# Supplementary material for: Comments on ”Evidence of the hydrogen release mechanism in bulk MgH2”
Source: Sci Rep. 2017 Apr 7;7:44216. doi: 10.1038/srep44216 (PMC5384078; doi:10.1038/srep44216)
Supplement: Supplementary Information [file srep44216-s2.pdf]

Supplementary Information

Comments on ”Evidence of the hydrogen release mechanism in bulk MgH_2” by K. Nogita et al.

Alexander Surrey[1,2,*], Kornelius Nielsch[1], and Bernd Rellinghaus[1]

[1] IFW Dresden, Institute for Metallic Materials, Dresden, D-01171 ,Germany
[2] Technische Universität Dresden, Institut für Festkörperphysik, Dresden, D-01062, Germany

[*] a.surrey@ifw-dresden.de

In-situ TEM heating

The video S1 shows the in-situ TEM heating of the Mg particle that was obtained through electron beam induced dehydrogenation during the former acquisition of SAD patterns (cf. Figure 1). The heating rate is 13K/min and the maximum temperature is T = 500 °C. Temperature and time stamp (format mm:ss) are indicated in the video. For this experiment we have used the Wildfire S3 heating holder from DENS solutions and the TEM images were acquired on a probe and image C_s corrected FEI Titan[3] 80-300 operated at 300 kV under low dose conditions with an electron current density of about 2500 electrons/s/nm^2.

SAD pattern of the particle after in-situ heating

The SAD pattern of the fully transformed particle at 500°C, which is shown in Figure 2d, is compared with a SAD pattern of commercial MgO nanopowder in Figure S2. The MgO nanopowder purchased from Sigma-Aldrich was dispersed on a carbon coated Cu TEM grid.

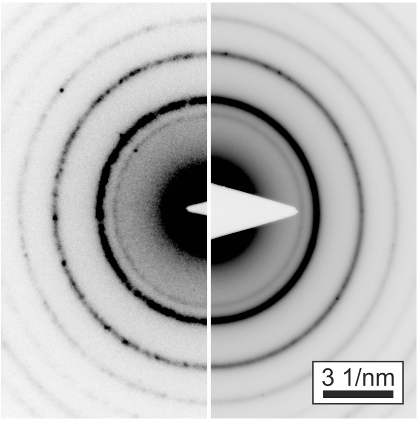

Figure S2. (left) SAD pattern of the fully transformed particle at 500°C. (right) SAD pattern of commercial MgO nanopowder.
